# Absorption mechanisms of Cu^2+^ on a biogenic bixbyite-like Mn_2_O_3_ produced by *Bacillus* CUA isolated from soil

**DOI:** 10.1186/s12932-015-0020-6

**Published:** 2015-05-13

**Authors:** Zhijun Zhang, Jing Lai, Hui Yin, Xionghan Feng, Wenfeng Tan, Fan Liu

**Affiliations:** Key Laboratory of Arable Land Conservation (Middle and Lower Reaches of Yangtse River), Ministry of Agriculture, College of Resources and Environment, Huazhong Agricultural University, Wuhan, 430070 China; CAS Key laboratory of Mineralogy and Metallogeny, Guangzhou Institute of Geochemistry, Chinese Academy of Sciences, Wushan, Guangzhou 510640 China

**Keywords:** Biogenic Mn oxide, Mn-oxidizing bacteria, Bixbyite, XAFS, Cu^2+^ adsorption

## Abstract

**Background:**

Although most reported biogenic Mn oxides are hexagonal birnessites, other types of biogenic Mn oxides also commonly occur in the environment. However, sorption characteristics and underlying mechanisms of the adsorption of heavy-metal ions on these biogenic Mn oxides are still rarely addressed. In this study, the sorption mechanisms of Cu(II) on a low valence biogenic Mn oxide, poorly crystallized bixbyite-like Mn_2_O_3_ (α-Mn_2_O_3_), were investigated.

**Results:**

The maximum adsorption capacity of Cu(II) onto this biogenic Mn oxide at pH 6.00 was 796 mmol/kg (0.45 mol Cu mol^−1^ Mn). The complex structure of adsorbed Cu(II) was constrained using Cu extended X-ray absorption fine structure (EXAFS) analysis, combined with structural parameters of the biogenic Mn oxide with alternately arranged regular and distorted MnO_6_ octahedra obtained through multiple-FEFF fitting of Mn EXAFS data. The sorbed Cu(II) was found to coordinate with the biogenic Mn oxide particle edges as inner-sphere complexes. At a relatively low Cu^2+^ loading (233 mmol/kg, pH 6.00), Cu(II) adsorbed onto the biogenic Mn oxide with two types of coordinated complexes, i.e., (1) coordinated with one regular/distorted MnO_6_ octahedron as a monodentate-mononuclear complex and (2) with two adjacent MnO_6_ octahedra as a bidentate-binuclear complex. While, at a relatively high Cu^2+^ loading (787 mmol/kg, pH 6.00), only one type of coordinated complex was constrained, the adsorbed Cu(II) coordinated with one regular/distorted MnO_6_ octahedron as a monodentate-mononuclear complex.

**Conclusions:**

This research extends further insight into the bacterial Mn(II) oxidation in the environment and serves as a good reference for understanding the interactions between metal ions and biogenic low valence Mn oxides, which are still poorly explored either theoretically or practically.

**Electronic supplementary material:**

The online version of this article (doi:10.1186/s12932-015-0020-6) contains supplementary material, which is available to authorized users.

## Background

Copper is one of the most ancient metals of human civilization, and a trace metal in most natural environments, posing no serious threat to biota and vegetation at its background levels. However, it often poses a threat to the environment in areas with elevated levels of copper as a result of contamination from, for example, mining of copper ore minerals, untreated industrial waste waters and its widespread use in agriculture, chemical and electronics industry [1–4]. Apart from organic matter [5, 6] and clay minerals [7], the chemical speciation and mobility of Cu(II) are linked in part to Mn oxides in soils and sediments [8–11]. Mn oxides are environmentally ubiquitous with multiple Mn oxidation states and an important source of reactive mineral surfaces in the environment. They usually exist as fine granular, spherical or film-like particles distributed in soils and sediments. As natural highly active sorbents and oxidants, Mn oxides widely participate in a variety of adsorption and/or redox reactions [12–14]. These reactions control the concentration, speciation, behavior and bioavailability of many heavy-metal ions and organic pollutants in the environment.

The formation of Mn oxides in the environment is generally considered to be closely related to the microbial activity. Up to now, microbially mediated Mn(II) oxidation processes have been studied mainly using three phylogenetically different bacteria: a marine *Bacillus* sp. Strain G-1, *Leptothrix discophora* strains SS-1 and SP-6, and *Pseudomonas putida* strains MnB1 and GB-1 [15–20] and a variety of fungi [13, 21–24]. Accordingly, our knowledge of the adsorption and oxidation of metal cations by biogenic Mn oxides and the transformation of biogenic Mn oxides into other Mn oxide minerals is mainly based on the Mn oxides produced by these microorganisms [12, 25–28]. However, the primary products of Mn(II) oxidation by the aforementioned microorganisms (bacteria and fungi) are exclusively nanoparticulate, poorly-crystalline hexagonal birnessites with the presence of Mn mainly as Mn(IV). Considering the diverse mechanisms of biooxidation of Mn(II) [20, 29], other types of biogenic Mn oxides could also be produced microbially in the environment. Hosseinkhani and Emtiati [30] first reported a gram-negative *Acinetobacter* sp. strain obtained from ocean water, the Mn(II)-oxidizing product of which was a bixbyite-like Mn_2_O_3_. Then, Zhang et al. [31] reported a *Bacillus* CUA isolated from soil, the Mn(II) oxidation product of which is also poorly crystallized bixbyite-like Mn_2_O_3_ (α-Mn_2_O_3_), a low valence of biogenic Mn oxides. However, compared to high valence biogenic Mn oxides (e.g., hexagonal birnessites), low valence biogenic Mn-oxides and especially their properties have been rarely reported, despite a plethora of studies on non-biogenic low valence Mn oxides. Webb et al. [32] suggested that Mn(III) is formed as an intermediate during the oxidation of Mn(II) by bacteria and can serve as both oxidant and reductant in one-electron-transfer reactions with other redox species in the biogeochemical processes. Therefore, the knowledge of formation, properties and surface reactivity of low valence biogenic Mn oxides could contribute to a new understanding of the biogeochemistry of Mn-oxides with various Mn valence states and the relevant elements (e.g., Cu(II)).

The sorption mechanisms of Cu(II) adsorbed to Mn oxides also has been extensively investigated [8, 11, 33–39]. Manceau et al. [35] claimed that the adsorbed Cu(II) on birnessite was six-fold coordination with four oxygen atoms at 1.96 Å and two at 2.23 Å, while, Sherman and Peacock [36] maintained that the adsorbed Cu(II) on birnessite was four-fold coordination. However, they are in agreement that the adsorbed Cu(II) coordinated with three surface oxygen atoms at vacancy sites of birnessite, forming a triple-corner-sharing complex with a Cu-Mn interatomic distance of 3.39–3.43 Å [35–37]. In addition, in aqueous solution, Cu(II) was reported to prefer a five-fold elongated square pyramidal coordination that is likely to be competitive with six-fold distorted octahedral coordination [40–42]. Sherman and Peacock [36] proposed that some Cu(II) was adsorbed in Cu-incorporation (Cu-INC) mode at a high pH value (pH ≈ 8.0), based on the appearance of a peak near 2.9 Å in the Fourier-transformed EXAFS spectrum for δ-MnO_2_ sample with a loading of 0.068 mol Cu mol^−1^ Mn. However, in a density functional theory (DFT) study, Kwon et al. [39] argued that the incorporation of Cu into a vacancy site strongly inhibits the Jahn-Teller distortion of Cu, destabilizing the Cu-INC species relative to Cu-TCS species at any pH value. More recently, Peña et al. [11] showed that Cu(II) was dominantly adsorbed at particle edges of δ-MnO_2_ as dimers or polynuclear surface species with a surface loading of 0.01 to 0.26 mol Cu mol^−1^ Mn.

However, previous studies on Cu(II) sorption onto Mn oxides are mainly focused on chemically synthesized layered Mn oxides, including some high-valence biogenic hexagonal birnessite. There are no reports about Cu(II) sorption onto low-valence biogenic Mn oxides and the issue of how the adsorbed Cu(II) coordinates with the Mn oxides is rarely addressed. Here, a low-valence biogenic Mn oxide was prepared, and the coordination mechanisms for the sorption of Cu(II), as a model of heavy metals, onto the biogenic Mn oxide were investigated using X-ray absorption spectroscopy (XAS).

## Results and discussion

### Sorption of Cu(II) onto the biogenic Mn oxide

**Fig. 1:**
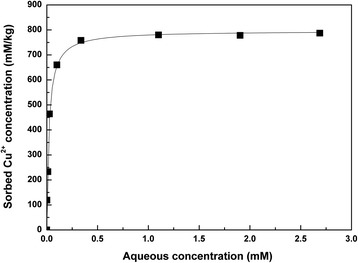
Sorption isotherm of Cu^2+^ on biogenic Mn oxide at 25 °C

Adsorption isotherm of Cu(II) on biogenic Mn oxide at pH6.0 is presented in Fig. 1. At the given pH, the final adsorbed amount (mmol/kg) increased with increasing initial Cu^2+^ concentration. The data were fitted to a Langmuir model with a correlation coefficient of R^2^ = 0.9904, indicating an adequate fit of the equation to the data. The maximum adsorption capacity was 796 mmol/kg (0.45 mol Cu mol^−1^ Mn). Therefore, the biogenic Mn oxide (α-Mn_2_O_3_) could also be a promising adsorbent for eliminating Cu(II) from soil and water environments. Two sorption samples, pH6.0-Cu0.4 and pH6.0-Cu4.0, as indicated by their initial pH and Cu^2+^ concentrations (mM), were used to collect EXAFS spectra. The adsorption densities of the two samples were 233 mmol/kg (0.13 mol Cu mol^−1^ Mn) for pH6.0-Cu0.4 and 787 mmol/kg (0.44 mol Cu mol^−1^ Mn) for pH6.0-Cu4.0, respectively.

### XAS analysis of sorbed Cu(II)

**Fig. 2:**
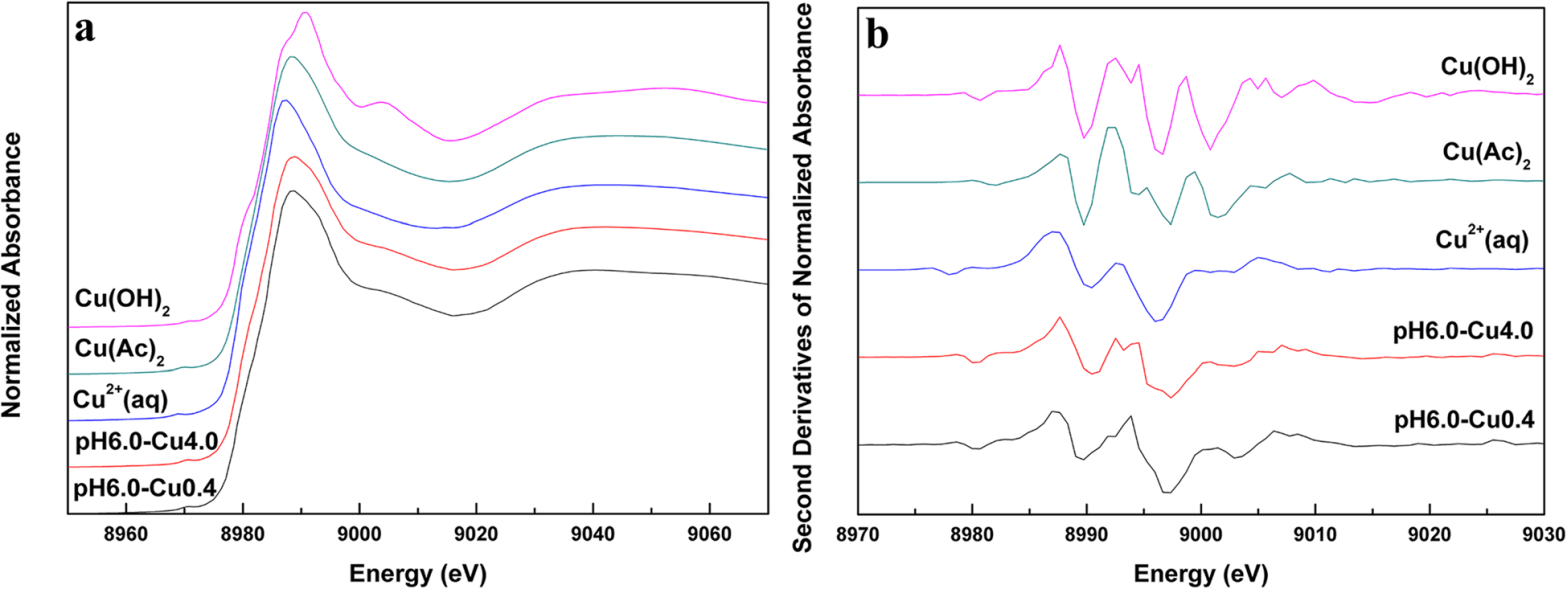
Normalized X-ray absorption edges and second derivatives of XANES of all samples and the reference compounds (**a**) normalized X-ray absorption edges of sorption samples and some reference compounds. **b** Second derivatives of XANES features of sorption samples and the reference compounds

**Fig. 3:**
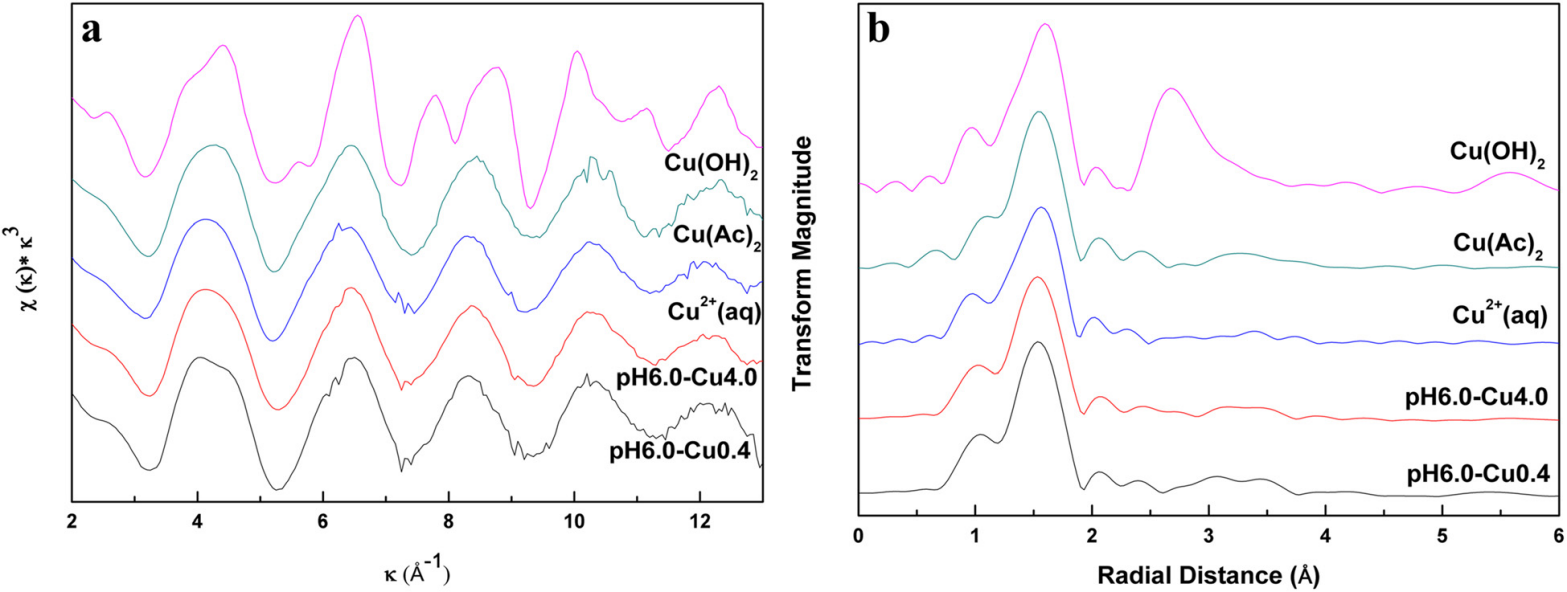
EXAFS results of the two samples with sorbed Cu(II) and reference compounds (**a**) K space spectra; (**b**) RSF spectra

Figure 2 shows X-ray absorption near-edge structure (XANES) spectra and second derivatives of the XANES spectra of the sorption samples and some reference compounds. The two sorption samples have similar X-ray absorption edges characteristic of divalent Cu(II) aqueous solution (Fig. 2a), indicating that the oxidation state of Cu on the surface of biogenic Mn oxide (Bixbyite) remains unchanged. In general, the sorbed Cu prefers to bind with Mn oxides, for example, birnessite, with three mechanisms: inner-sphere surface at cation vacancy sites and/or particle edges, incorporation into the MnO_2_ sheet and Cu polymerization or precipitation [11]. The two sorption samples differ from Cu(OH)_2_ not only in the shape and position of the XANES spectra, but also in second derivative (Fig. 2b) and EXAFS (Fig. 3), thereby eliminating the possibility of polymerization or precipitation. In tetrahedral coordination, the pre-edge feature in Cu K-edge XANES should show greater intensity and be shifted towards slightly lower energies [11, 43, 44], which meant that the sorbed Cu(II) was not in tetrahedral coordination. The second derivative of copper acetate monohydrate, a model compound with isolated dimeric Cu units, was used to determine whether dimeric Cu units were present in sorbed Cu(II) [45]. Here, the two sorption samples have second derivative differ with copper acetate monohydrate, which means no dimeric Cu unit exist in the sorbed Cu(II) on the surface of biogenic Mn oxides.

Figure 3 shows EXAFS and Fourier transforms (FT) of the sorption samples and those of reference compounds. Generally, the d^9^ electronic configuration of Cu(II) leads to the Jahn-Teller distortion of the first coordination shell and this structure distortion leads to two distinct Cu-O shell subshells, where the equatorial sub-shell dominates the EXAFS signal, but no reliable coordination numbers and distances of axial sub-shell could be fitted due to the anisotropic disorder [11, 45, 46]. Thus, we fit the first Cu-O shell EXAFS spectra of Cu^2+^ (aq) with coordination number fixed at 4 and got the amplitude reduction factor (S_0_^2^ = 0.96). Then, the other EXAFS spectra were fitted with S_0_^2^ set at 0.96, and the fitting results are listed in Table 1. It shows that the main EXAFS contributions of the reference compounds are from oxygen atoms at approximately ~1.95 Å, with coordination number close to 4 of this first Cu-O shell, which agrees well with several previous reports [11, 36, 45]. Additionally, all of the Cu-O shells with a low Debye-Waller factor (DWF, 0.005–0.007 Å^2^), indicates a low local structural disorder and these Cu-O bonds are the typical equatorial bonds of the distorted Cu polyhedron. Manceua et al. [35] obtained an improved fit by 4 equatorial oxygen atoms at 1.96 Å and two axial oxygen atoms at 2.23 Å, with a much larger DWF, as expected for Cu in a Jahn-Teller distorted environment. We cannot confirm whether Cu has one or two axial ligands in our sorption samples, due to no obvious improvement with the addition of one or two axial oxygen atoms. However, in principle, both the results are reasonable, because Cu in aqueous solution prefers a five-fold coordination that is likely to be competitive with a six-fold distorted octahedral coordination [36, 40–42].

**Table 1:** Fitting results of the EXAFS spectra of Cu^2+^ sorbed on biogenic manganese oxide

Sample	Cu-O			Cu-Mn		
	R(Å)^a^	CN^b^	σ^2^(Å^2^)^c^	R(Å)^a^	CN^b^	σ^2^(Å^2^)^c^
Cu^2+^(aq)	1.953	4.0^d^	0.007	-	-	-
	(0.001)		(0.001)			
Cu(OH)_2_	1.948	4.1	0.005	-	-	-
	(0.001)	(0.3)	(0.001)			
Cu(Ac)_2_	1.946	4.0	0.005			
	(0.001)	(0.2)	(0.001)			
pH6.0-Cu0.4	1.945	4.2	0.006	3.371	1.7	0.017
	(0.001)	(0.3)	(0.001)	(0.001)	(0.3)	(0.009)
pH6.0-Cu4.0	1.944	4.0	0.006	3.440	0.7	0.019
	(0.001)	(0.1)	(0.001)	(0.001)	(0.3)	(0.010)

^a^Interatomic distance

^b^Coordination number

^c^the Debye-Waller factor, with in brackets the average error

^d^Number fixed during fit

**Fig. 4:**
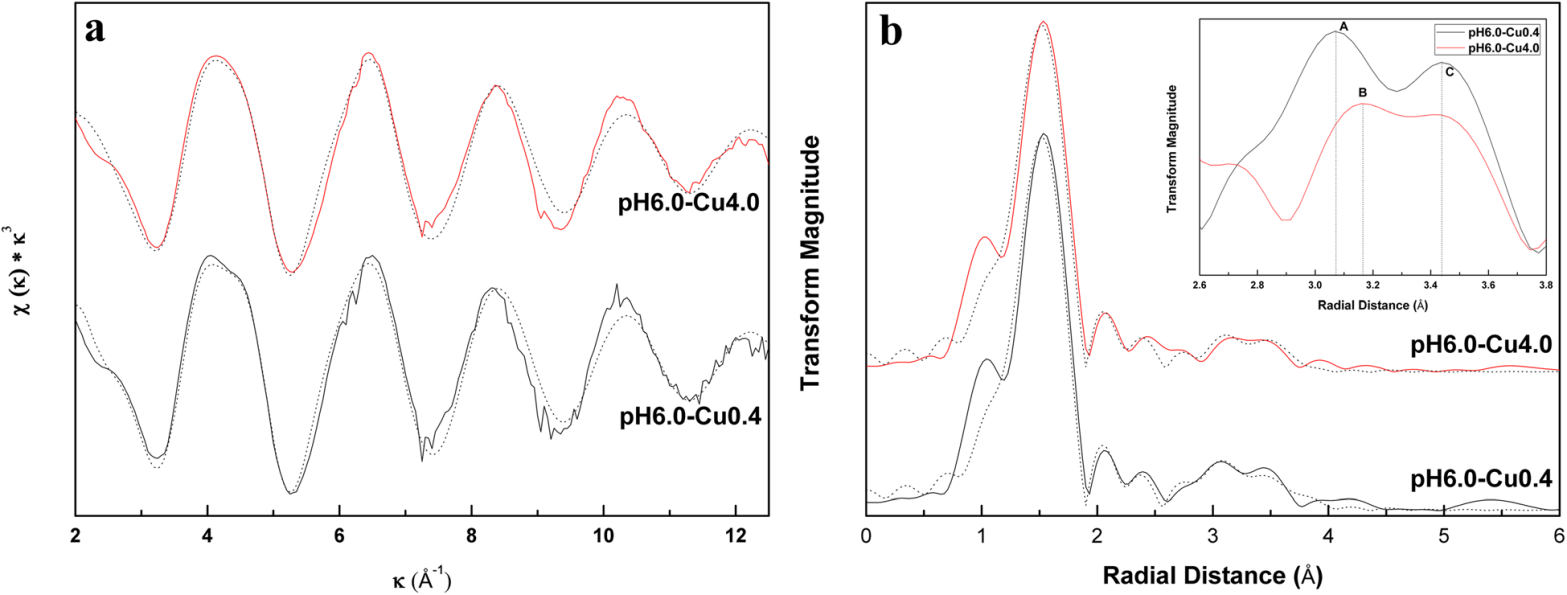
EXAFS fitting results of the two samples with sorbed Cu(II) (**a**) K space spectra; (**b**) RSF spectra; *inset*: RSFs of the samples in the *R*-range of 2.6–3.8 Å. (EXAFS data: *solid line*; model fitting: *dotted line*)

For the higher shell of the sorption samples, there are two main characteristic peaks (Fig. 4b), peak A (~3.07 Å, phase shift uncorrected) of pH6.0-Cu0.4, peak B (~3.14 Å) of pH6.0-Cu4.0 and peak C (~3.44 Å) of the two samples. The final fitting of the raw data showed that the best fit is with one shell of oxygen at about 1.95 Å, one shell of Mn at about 3.40 Å, and a Cu-O_eq_-O_eq_ multiple scattering path at about 3.90 Å, which is approximately twice the distance of the Cu-O_eq_ shell (1.95 Å) [45]. The fitting results are given in Fig. 4 and Table 1.

Table 1 shows that the Cu-Mn distance of the sample pH6.0-Cu0.4 is 3.37 Å with a coordination number of 1.7 (0.3) and that of the sample pH6.0-Cu4.0 is 3.44 Å with a coordination number of 0.7 (0.3). Sherman and Peacock [36] showed that the Cu-Mn interatomic distance of sobred Cu incorporation into the MnO_2_ sheet was in the range of 2.81–2.94 Å. In a density functional theory study, Kown et al. [39] showed that the theoretical values of Cu-Mn distance in incorporation mode are 2.84 Å, 2.95 Å and 2.98 Å. Though the biogenic Mn oxide (Bixbyite) in this study differs in structure with birnessite, the Cu-Mn distance (3.37 Å and 3.44 Å) in the two sorption samples should be unlikely in incorporation mode. Thus, we deny the possibility of Cu incorporation into the biogenic Mn oxide in our study. In birnessite, the adsorption Cu(II) at cation vacancy sites involves the coordination of the copper to the three surface oxygen atoms surrounding the vacancy site, thus forming a triple-corner-sharing (TCS) complex [11, 35, 47]. The Cu-Mn interatomic distances of Cu-TCS complexes in birnessite were inferred in the range of 3.39–3.43 Å with a Cu-Mn shell coordination number of 3 [35–37]. Based on the Cu-Mn distance, the sorbed Cu(II) on the two samples could be in Cu-TCS complexes, but the coordination numbers of the two sorption samples are far less than 3. Additionally, there is no report of cation vacancy site in the structure of bixbyite [31]. Therefore, the sorbed Cu(II) on the two samples are unlikely in Cu-TCS complexes. Based on the above analysis, the most possible way of sorbed Cu(II) on the biogenic Mn oxide is coordinated with the particle edges as inner-sphere surface complexes. To find out the specific ways in which the sorbed Cu(II) coordinated with the MnO_6_ octahedra, we used the EXAFS analysis results of our samples and the polyhedral approach for further investigation.

### Constraints on the structure of Cu^2+^ complexes on the low valence biogenic Mn oxide

**Fig. 5:**
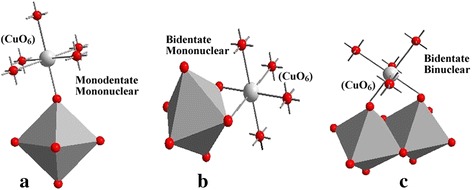
Three types of linkage between sorbed Cu^2+^ (CuO_6_ octahedron) and the MnO_6_ octahedron on the surface of biogenic Mn oxide: (**a**) monodentate mononuclear (*MM*); (**b**) bidentate mononuclear (*BM*); (**c**) bidentate binuclear (*BB*)

We cannot confirm whether Cu has one or two axial ligands in our sorption samples, but, in principle, both the results are reasonable [11, 39], because the five-fold-coordinate Cu complex showed one very long axial Cu-O distance of Cu octahedron, such that the Cu coordination appeared to be essentially five-fold [39]. Kwon et al. [39] also showed in their DFT calculation that the structural parameters of the five-fold-coordinate complex were not distinguishable from the corresponding parameters of the octahedral complex. Thus, we only used Cu octahedron in our following geometric analysis. The ligand bond to Cu(II) octahedron could be O, OH, or H_2_O, and we will use O throughout this paper to represent these possible ligands. The equatorial oxygen-oxygen (O-O) distances in a distorted Cu(II)O_6_ octahedron are approximately 2.80 Å [45], and the Cu-O distances of our adsorption samples are approximately 1.95 Å (Table 1). The Mn-O distances of MnO_6_ in the biogenic Mn oxide are 2.095 Å, 1.903 Å, 2.178 Å and 2.350 Å, respectively [31]. Based on these coordination distances and the geometry coordination model (Fig. 5), we analyzed the possible linkages between a Cu(II)O_6_ octahedron and a MnO_6_ octahedron as follows:

1. A Cu(II)O_6_ octahedron and one MnO_6_ octahedron can link as a monodentate mononuclear (MM) complex (Fig. 5a). This structure could exhibit Cu-Mn distances in the range of approximately 2.800–4.300 Å. The range of Cu-Mn distances is large because Cu can be coordinated by either equatorial or axial oxygen. Furthermore, it is theoretically possible that a Cu(II)O_6_ octahedron and a MnO_6_ octahedron could link at a flexible angle. The Cu-Mn distances observed in the two samples (Table 1) are all within the indicated range, suggesting that MM is the first likely linkage.
2. A Cu(II)O_6_ octahedron and one MnO_6_ octahedron can link as a bidentate mononuclear (BM) complex (Fig. 5b). The Cu-Mn distance in this edge-sharing mode is approximately 2.80 Å, which is much shorter than the Cu-Mn distances in the two samples (3.37 Å, 3.44 Å), implying that the possibility of a face-sharing and an edge-sharing linkage can be excluded.
3. A Cu(II)O_6_ octahedron and two adjacent MnO_6_ octahedra can link as a bidentate binuclear (BB) complex (Fig. 5c). Based on a spatial geometry, the theoretical Cu-Mn distance for this BB linkage can be calculated using the following trigonometric function:
  $$ d{\left(\mathrm{C}\mathrm{u}\hbox{-} \mathrm{M}\mathrm{n}\right)}^2=d{\left(\mathrm{C}\mathrm{u}\hbox{-} \mathrm{O}\right)}^2+d{\left(\mathrm{M}\mathrm{n}\hbox{-} \mathrm{O}\right)}^2-2d\left(\mathrm{C}\mathrm{u}\hbox{-} \mathrm{O}\right)\times d\left(\mathrm{M}\mathrm{n}\hbox{-} \mathrm{O}\right)\times \cos \left(\theta \right), $$

where *d* is the atomic distance of the indicated interatomic bond, and *θ* is the Mn-O-Cu angle. The Cu-Mn distance in this mode is approximately in the range of 2.73 to 3.98 Å (Additional file 1: S1). The Cu-Mn distances of the two samples (Table 1) are all within the indicated range, implying that BB is the second likely linkage. However, the coordination number of the sample pH6.0-Cu4.0 is 0.7 (0.3), which is far less than the theoretical coordination number (*CN* = 2) of BB mode, indicating that the possibility of a bidentate binuclear (BB) complex in the sample pH6.0-Cu4.0 can be rejected.

**Fig. 6:**
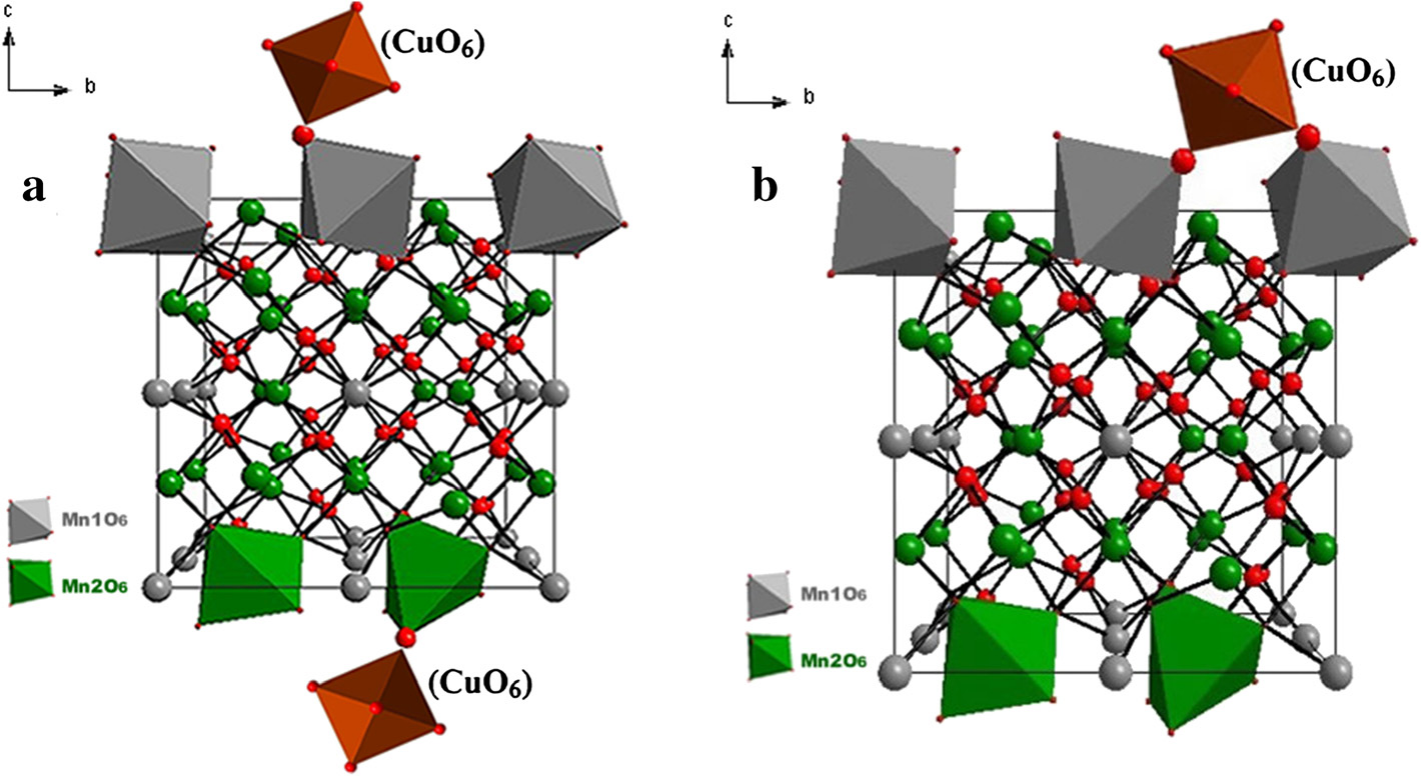
The final possible linkage between the CuO_6_ octahedron and the MnO_6_ octahedron of biogenic Mn oxide (**a**) one CuO_6_ octahedron and one MnO_6_ octahedron, regular (*grey*) or distorted (*green*), linked as MM complex; (**b**) one CuO_6_ octahedron and two regular MnO_6_ octahedra linked as BB complex

Therefore, at a low Cu^2+^ loading, the sorbed Cu^2+^ could link with the MnO_6_ octahedra with two types of complexes: (1) one CuO_6_ octahedron and one MnO_6_ octahedron (regular or distorted) linked as MM (Fig. 6a); (2) one CuO_6_ octahedron and two adjacent MnO_6_ octahedra linked as BB (Fig. 6b). At a high Cu^2+^ loading, Cu^2+^ (CuO_6_) could link with MnO_6_ octahedra only in one possible type of complex, one CuO_6_ octahedron and one MnO_6_ octahedron (regular or distorted) linked as MM (Fig. 6a). There is a little difference in the coordination mode between the low and high Cu^2+^ loading samples, probably because there are still some adsorption sites in the low Cu^2+^ loading sample (233 mmol/kg), resulting in the shift of some sorbed Cu^2+^ in the monodentate mononuclear (MM) complex to the bidentate binuclear (BB) complex, a more stable geometric structure. However, in the high Cu^2+^ loading sample (787 mmol/kg), there is almost no additional adsorption site in it, because the adsorption density (787 mmol/kg) is almost in the maximum adsorption capacity (796 mmol/kg) of the biogenic Mn oxide.

It should be noted that, in all of the above analyses, mainly the bulk structure of the biogenic Mn oxide (bixbyite) was considered, implying that the bulk and the surface structure of the biogenic Mn oxide were assumed to be the same. When the surface structure becomes available, the options for the Cu^2+^ complex structures on the surface of the biogenic bixbyite-like phase might be further constrained. The aforementioned results could provide a good reference for understanding the interactions between metal cations and biogenic or abiogenic low-valence Mn oxides, which are still poorly explored both theoretically and experimentally.

### Experimental

#### Biogenic Mn oxide

The biogenic Mn oxide was reported in our previous work [31]. Briefly, biogenic Mn oxides were produced by culturing an isolated Mn-oxidizing bacterium using *Leptothrix discophora* medium with initial Mn(II) concentration at 1.0 mM. The biogenic Mn oxide was harvested after 12 days via centrifugation at 10,000 RCF (25 °C, 5 min), because at which time the Mn-oxide concentration was at its maximum. The biogenic Mn oxide used in this study is a poorly-crystalline bixbyite (JCPDS 01–078–0390), with a bulk-averaged Mn oxidation state (AOS) of 2.73. It is composed of uniform aggregates of granular particles with sizes of 25–50 nm, and a specific surface area of 49 m^2^/g [31].

Bixbyite is a cubic, granular manganese oxide mineral of the cubic crystal system *Ia-3* (SG.NO.206) with regular and distorted MnO_6_ octahedra alternately arranged in the structure [48, 31]. In the structure of crystalline bixbyite, the cations occupy the 8*a* site at 0, 0, 0, etc. and the 24*d* site at x, 0, 0.25, etc., with x = 0.2855 [48]. The anions occupy the general 48*e* site, x, y, z (0.1293, 0.1471, −0.0835), whereas the 16*c* site, x, x, x, is unoccupied, being the site of the “anion vacancies”, with x = 0.125. The cell formula is Mn_32_O_48_□_16_ (□ denotes an anion vacancy) [48]. In the ideal structure of bixbyite, the Mn1 atoms of the 8*a* site are incorporated into the regular Mn1O_6_ octahedra and the Mn2 atoms of the 24*d* site are part of the highly distorted Mn2O_6_ octahedra/polyhedron due to the distortion of the 3d^4^ Mn(III) ions. The six Mn-O distances of the regular Mn1O_6_ octahedra in the prepared biogenic Mn oxide of this study are 2.095 Å, while the Mn-O distances in the distorted Mn2O_6_ octahedra have three different pairs at 1.903 Å, 2.178 Å and 2.350 Å [31].

### Sorption of Cu^2+^ on biogenic Mn Oxide

Sorption experiments of Cu^2+^ on the biogenic Mn oxides were carried out as previously reported by Zhao et al. [49] and Zhang et al. [31]. Briefly, 5.0 g/L mineral suspensions were prepared and equilibrated for a few days, during which the pH was adjusted to 6.00 (±0.05) with 0.1 M HNO_3_ or 0.1 M NaOH. Sorption experiments were initiated by adding 20 mL of the final suspension to a 100-mL polypropylene bottle containing 0.8 mL to 16 mL of 15-mM Cu(NO_3_)_2_ solution. The total volume of the solution in each bottle was increased to 60 mL by adding 0.15 M NaNO_3_. Therefore, the sorbent concentration was 1.67 g/L, the ionic strength was approximately 0.1 M and the Cu^2+^ initial concentrations were 0.2 to 4.0 mM, respectively. The polypropylene bottles with suspensions were immediately capped and shaken, and the pH was adjusted twice during the equilibration period to 6.00 (±0.05). After 24 h of equilibration, the suspensions were centrifuged. Approximately 10 mL of the clear supernatant liquid was used for the determination of the Cu^2+^ concentration using a Varian AAS 240FS atomic absorption spectrometer (USA). The precipitates were collected and conserved at 4 °C for the XAS analysis.

### XAS data collection

The XAS spectra of all of the samples were collected on the 1W1B beamline at Beijing Synchrotron Radiation Facility (BSRF) [31, 50–52]. Cu sorption samples were obtained from centrifugation and prepared by the procedures of Zhang et al. [31]. The EXAFS spectra (8979 eV) were collected with a Lytle ion-chamber detector in fluorescence mode. The reference compounds, solid Cu(OH)_2_ powder, copper acetate monohydrate and copper aqueous solutions (0.1 M Cu(NO_3_)_2_) were measured in transmission mode. The energy was calibrated with Cu foil. Reduction and analysis of all XAS data were performed using IFEFFIT/SIXPACK [53]. The Cu *K*-edge averaged spectra were background-subtracted using the following parameters: *E*_0_ = 8979 eV, *Rbkg* = 1.0 Å and *k*-weight = 3. Phase and amplitude functions for single-scattering paths were calculated using FEFF7 [54]. An amplitude reduction factor (S_0_^2^) of 0.96 was determined by fitting the Cu-O shell of Cu(NO_3_)_2_(aq) spectra in the *R*-range 1.0–2.0 Å, and the other samples were fitted with S_0_^2^ fixed at 0.96. The calculations of the phase shift and amplitude functions of Cu-Mn were based on the crystal structure of CuMnO_2_ (Crednerite). Final fits of the two sorption samples were made in Artemis in *R*-range 1.0–3.8 Å.

## Conclusions

In this study, a low valence biogenic Mn oxide, a poorly crystallized bixbyite-like Mn_2_O_3_ (α-Mn_2_O_3_), which has barely been studied in the past several decades, was used as an adsorbent and the adsorption mechanisms of Cu(II) on it were studied. The maximum adsorption capacity of Cu(II) adsorbed to the biogenic Mn oxide at pH 6.00 was 796 mmol/kg, indicating that the biogenic Mn oxide could be an promising adsorbent for eliminating Cu(II) from soil and water environments. At a relatively low Cu(II) loading (233 mmol/kg) sample, Cu(II) was adsorbed onto the biogenic Mn oxide as inner-sphere complexes and linked with MnO_6_ octahedra by two types of complexes: MM and BB. At a relatively high Cu(II) loading (787 mmol/kg), the adsorbed Cu(II) linked with MnO_6_ octahedra only by MM type of complex. These results provide further insight into the bacterial Mn(II) oxidation in the environment and a good reference for understanding the biogeochemistry of low valence of biogenic Mn oxides and their interactions with metal ions in the natural environments, which are still poorly explored, either theoretically or practically. Furthermore, this research could also enrich or contribute a new understanding of the biogeochemical processes of Mn oxides and their environmental significance.

## Additional file

Additional file 1:
**S1 Calculation of the possible Cu-Mn distance of BB. **The following additional data are available with the online version of this paper.

## References

[1] Grimalt JO, Ferrer M, Macpherson E (1999). The mine tailing accident in Aznalcollar. Sci Total Environ.

[2] Younger PH (2000). Nature and practical implications of heterogeneities in the geochemistry of zinc-rich, alkaline mine waters in an underground F-Pb mine in the UK. Appl Geochem.

[3] Rico A, Benito G, Diez-Herrero A (2008). Floods from tailings dam failures. J Hazard Mater.

[4] Zhuang P, McBride MG, Xia HP, Li NY, Lia ZA (2009). Health risk from heavy metals via consumption of food crops in the vicinity of Dabaoshan mine, South China. Sci Toal Environ.

[5] Jacobson AR, Dousset S, Andreux F, Baveye PC (2007). Electron microprobe and synchrotron X-ray fluorescence mapping of the heterogeneous distribution of copper in high-copper vineyard soils. Environ Sci Technol.

[6] Strawn DG, Baker LL (2008). Speciation of Cu in a contaminated agricultural soil measured by XAFS, μ-XRF. Environ Sci Technol.

[7] Hochella MF, Moore JN, Putnis CV, Putnis A, Kasama T, Eberl DD (2005). Direct observation of heavy metal mineral association from the Clark Fork River superfund complex: implications for metal transport and bioavailability. Geochimica Et Cosmochimica Acta.

[8] Jenne EA, Baker RA (1968). Controls on Mn Fe Co Ni Cu and Zn concentrations in soils and water: the significant role of hydrous Mn and Fe Oxides. Trace inorganics in water. Vol. 73.

[9] McLaren RG, Crawford DV (1973). Studies on soil copper .II. Specific adsorption of copper by soils. J Soil Sci.

[10] Davies-Colley RJ, Nelson PO, Williamson KJ (1984). Copper and cadmium uptake by estuarine sedimentary phases. Environ Sci Technol.

[11] Peña J, Bargar JR, Sposito G (2015). Copper sorption by the edge surfaces of synthetic birnessite nanoparticles. Chem Geol.

[12] Feng XH, Zhu MQ, Ginder-Vogel M, Ni CY, Parikh SJ, Sparks DL (1974). Formation of nano-crystalline todorokite from biogenic Mn oxides. Geochimica Et Cosmochimica Acta.

[13] Santelli CM, Webb SM, Dohnalkova AC, Hansel CM (2011). Diversity of Mn oxides produced by Mn(II)-oxidizing fungi. Geochimica Et Cosmochimica Acta.

[14] Zhu MQ, Farrow CL, Post JE, Livi KJT, Billinge SJL, Ginder-Vogel M (2012). Structural study of biotic and abiotic poorly-crystalline manganese oxides using atomic pair distribution function analysis. Geochimica Et Cosmochimica Acta.

[15] Mandernack KW, Post J, Tebo BM (1995). Manganese mineral formation by bacterial spores of the marine Bacillus, strain SG-1: evidence for the direct oxidation of Mn(II) to Mn(IV). Geochimica Et Cosmochimica Acta.

[16] Tebo BM, Ghiorse WC, van Waasbergen LG, Siering PL, Caspi R, Banfield JF, Nealson KH (1997). Bacterially mediated mineral formation: insights into manganese(II) oxidation from molecular genetic and biochemical studies. Geomicrobiology: interactions between microbes and minerals. Reviews in mineralogy. Vol. 35.

[17] Caspi R, Tebo BM, Haygood MG (1998). c-Type cytochromes and manganese oxidation in Pseudomonas putida MnB1. Appl Environ Microbiol.

[18] De Vrind JPM, Brouwers GJ, Corstjens PLAM, den Dulk J, de Vrind-de Jong EW (1998). The cytochrome c maturation operon is involved in manganese oxidation in Pseudomonas putida GB-1. Appl Environ Microbiol.

[19] Bargar JR, Tebo BM, Villinski JE (2000). In situ characterization of Mn(II) oxidation by spores of the marine Bacillus sp. strain SG-1. Geochimica Et Cosmochimica Acta.

[20] Tebo BM, Bargar JR, Clement BG, Dick GJ, Murray KJ, Parker D (2004). Biogenic manganese oxides: properties and mechanisms of formation. Annu Rev Earth Planet Sci.

[21] Tanaka K, Tani Y, Takahashi Y, Tanimizu M, Suzuki Y, Kozai N (2010). A specific Ce oxidation process during sorption of rare earth elements on biogenic Mn oxide produced by Acremonium sp. strain KR21-2. Geochimica Et Cosmochimica Acta.

[22] Peña J, Kwon KD, Refson K, Bargar JR, Sposito G (2010). Mechanisms of nickel sorption by a bacteriogenic birnessite. Geochimica Et Cosmochimica Acta.

[23] Takano K, Itoh Y, Ogino T, Kurosawa K, Sasaki K (2006). Phylogenetic analysis of manganese-oxidizing fungi isolated from manganese-rich aquatic environments in Hokkaido, Japan. Limnology.

[24] Yu QQ, Sasaki K, Tanaka K, Ohnuki T, Hirajima T (2012). Structural factors of biogenic birnessite produced by fungus Paraconiothyrium sp.WL-2 strain affecting sorption of Co^2+^. Chem Geol.

[25] Nelson YM, Lion LW, Ghiorse WC, Shuler ML (1999). Production of biogenic Mn oixdes by leptothrix descophora SS-1 in a chemically defined growth medium and evaluation of their Pb adsorption characteristics. Appl Environ Microbiol.

[26] Villalobos M, Bargar J, Sposito G (2005). Mechanisms of Pb(II) sorption on a biogenic manganese oxide. Environ Sci Technol.

[27] Miyata N, Maruo K, Tani Y, Tsuno H, Seyama H, Soma M (2006). Production of biogenic manganese oxides by anamorphic Ascomycete fungi isolated from streambed pebbles. Geomicrobiol J.

[28] Zhu MQ, Ginder-Vogel M, Parikh SJ, Feng XH, Sparks DL (2010). Cation effects on the layer structure of biogenic Mn-Oxides. Environ Sci Technol.

[29] Learman DR, Voelker BM, Vazquez-Rodriguez AI, Hansel CM (2011). Formation of manganese oxides by bacterially generated superoxide. Nature Geo.

[30] Hosseinkhani B, Emtiati G (2011). Synthesis and characterization of a novel extracellular biogenic manganese oxide (bixbyite-like Mn_2_O_3_) nanoparticle by isolated acinetobacter sp. Curr Microbiol.

[31] Zhang ZJ, Yin H, Tan WF, Koopal LK, Zheng LR, Feng XH (2014). Zn sorption to biogenic bixbyite-like Mn_2_O_3_ produced by *Bacillus* CUA isolated from soil: XAFS study with constraints on sorption mechanism. Chem Geol.

[32] Webb SM, Dick GJ, Bargar JR, Tebo BM (2005). Evidence of the presence of Mn(III) intermediates in the bacterial oxidation of Mn(II). Proc Natl Acad Sci U S A.

[33] Traina SJ, Doner HE (1985). Copper manganese(II) exchange on a chemically reduced birnessite. J Soil Sci Soc Am.

[34] Karthikeyan KG, Elliott HA, Chorover J (1999). Role of surface precipitation in copper sorption by the hydrous oxides of iron and aluminum. J Colloid Interface Sci.

[35] Manceau A, Lanson B, Drits VA (2002). Structure of heavy metal sorbed birnessite. Part III: results from powder and polarized extended X-ray absorption fine structure spectroscopy. Geochimica Et Cosmochimica Acta.

[36] Sherman DM, Peacock CL (2010). Surface complexation of Cu on birnessite (delta-MnO2): controls on Cu in the deep ocean. Geochimica Et Cosmochimica Acta.

[37] Arai Y (2011). Aqueous interfacial chemistry of kaolinite for the removal of Cu(II) in the presence of birnessite: kinetic and spectroscopic studies. Appl Clay Sci.

[38] Wang Y, Feng XH, Villalobos M, Tan WF, Liu F (2012). Sorption behavior of heavy metals on birnessite: relationship with its Mn average oxidation state and implications for types of sorption sites. Chem Geol.

[39] Kwon KD, Refson K, Sposito G (2013). Understanding the trends in transition metal sorption by vacancy sites in birnessite. Geochimica Et Cosmochimica Acta.

[40] Pasquarello A, Petri I, Salmon PS, Parisel O, Car R, Tóth E (2001). First solvation shell fo the Cu(II) aqua ion: evidence for fivefold coordination. Science.

[41] Frank P, Benfatto M, Szilagyi RK, D’Angelo P, Della Longa S, Hodgson KO (2005). The solution structure of [Cu(aq)]^2+^ and its implications from rack-induced bonding in blue copper protein active. Inorg Chem.

[42] Bryantsev VS, Diallo MS, van Duin ACT, Goddard WA (2008). Hydration of copper(II): New insights from density functional theory and the COSMO solvation model. J Phys Chem.

[43] Shimizu K, Maeshima H, Yoshida H, Satsuma A, Hattori T (2001). Ligand field effect on the chemical shift in XANES spectra of Cu(II) compounds. Phys Chem Chem Phys.

[44] Dupont L, Guillon E, Bouanda J, Dumonceau J, Aplincourt M (2002). EXAFS and XANES studies of retention of copper and lead by a lignocellulosic biomaterial. Environ Sci Technol.

[45] Cheah SF, Brown GE, Parks GA (1998). XSFS spectroscopy study of Cu(II) sorption on amorphous SiO_2_ and γ-Al_2_O_3_: effect of substrate and time on sorption complexes. J Colloid Interface Sci.

[46] Cheah SF, Brown GE, Parks GA (2000). XAFS study of Cu model compounds and Cu^2+^ sorption products on amorphous SiO2, gamma-Al_2_O_3_, and anatase. Am Mineral.

[47] Manceau A, Lanson M, Geoffroy N (2007). Natural speciation of Ni, Zn, Ba, and As in ferromanganese coatings on quartz using X-ray fluorescence, absorption, and diffraction. Geochimica Et Cosmochimica Acta.

[48] Bevan DJM, Martin RL (2000). The role of the coordination defect: a new structural description of four fluorite-related sesquioxide minerals, bixbyite (Mn_2_O_3_), braunite (Mn_7_SiO_12_), braunite (CaMn_14_SiO_24_), parwelite (Mn_10_Sb_2_As_2_Si_2_O_24_), and their structural relationships. J Solid State Chem.

[49] Zhao W, Cui HJ, Liu F, Tan WF, Feng XH (2009). Relationship between Pb^2+^ adsorption and average Mn oxidation state in synthetic birnessites. Clays Clay Miner.

[50] Yin H, Tan WF, Zheng LR, Cui HJ, Qiu GH, Liu F (2012). Characterization of Ni-rich hexagonal birnessite and its geochemical effects on aqueous Pb^2+^/Zn^2+^ and As(III). Geochimica Et Cosmochimica Acta.

[51] Yin H, Liu F, Feng XH, Hu TD, Zheng LR, Qiu GH (2013). Effects of Fe doping on the structures and properties of hexagonal birnessites-Comparison with Co and Ni doping. Geochimica Et Cosmochimica Acta.

[52] Yin H, Li H, Wang Y, Ginder-Vogel M, Qiu GH, Feng XH (2014). Effects of Co and Ni co-doping on the structure and reactivity of hexagonal birnessite. Chem Geol.

[53] Webb SM (2005). SIXpack: a graphical user interface for XAS analysis using IFEFFIT. Phys Scr.

[54] Rhehr JJ, Zabinsky SI, Albers RC (1992). High-order multiple scattering calculations of X-ray-absorption fine structure. Phys Rev Lett.

