# Supplementary material for: Absorption mechanisms of Cu2+ on a biogenic bixbyite-like Mn2O3 produced by Bacillus CUA isolated from soil
Source: Geochem Trans. 2015 May 13;16:5. doi: 10.1186/s12932-015-0020-6 (PMC4434580; doi:10.1186/s12932-015-0020-6)
Supplement: Additional file 1: — S1 Calculation of the possible Cu-Mn distance of BB. The following additional data are available with the online version of this paper. [file 12932_2015_20_MOESM1_ESM.doc]

***Supplementary material***

**Absorption mechanisms of Cu^2+^ on a biogenic bixbyite**-**like Mn_2_O_3_ produced by**

***Bacillus* CUA isolated from soil**

Zhijun Zhang^1^, Jing Lai^2^, Hui Yin^1^, Xionghan Feng^1^, Wenfeng Tan^1^, Fan Liu ^1^,*

^1^ Key Laboratory of Arable Land Conservation (Middle and Lower Reaches of Yangtse River), Ministry of Agriculture, College of Resources and Environment, Huazhong Agricultural University, Wuhan 430070, China

Zhijun Zhang: [zhijun@webmail.hzau.edu.cn](mailto:zhijun@webmail.hzau.edu.cn)

Hui Yin: [yinhui666@mail.hzau.edu.cn](mailto:yinhui666@mail.hzau.edu.cn)

Xionghan Feng: [fxh73@mail.hzau.edu.cn](mailto:fxh73@mail.hzau.edu.cn)

Wenfeng Tan: [wenfeng.tan@hotmail.com](mailto:tanwf@mail.hzau.edu.cn)

Fan Liu: [liufan@mail.hzau.edu.cn](mailto:liufan@mail.hzau.edu.cn)

^2^ CAS Key laboratory of Mineralogy and Metallogeny, Guangzhou Institute of Geochemistry, Chinese Academy of Sciences, Wushan, Guangzhou 510640, China

Jing Lai: [laijing@gig.ac.cn](mailto:laijing@gig.ac.cn)

*Corresponding author:

E-mail: [liufan@mail.hzau.edu.cn](mailto:liufan@mail.hzau.edu.cn) (F. Liu)

Tel: 027-87280271; Fax: +86 27 87288618

S1 Calculation of the possible Cu-Mn distance of BB

In the bidentate binuclear (BB) complex (Fig. 5c), we fixed the Mn-O-Zn angle at 130° in our previous work of Zn(II) sorbed on this biogenic Mn oxide [1], because of Pan et al (2004) [2] reported it. However, no reported about the angle of sorbed Cu(II) on Mn oxides at this complex, and here, we just analyse from an ideal geometry configuration. The maximum value of Cu-Mn distance is when the Cu atom, two sharing O atoms and the Mn atom in the same plane, which means that the Mn-O-Cu angle is 135° in the ideal configuration. So, the maximum theoretical value of Cu-Mn distance can be calculated using the following trigonometric function:

*d*(Cu-Mn)^2^ = *d*(Cu-O)^2^ + *d*(Mn-O)^2^ – 2*d*(Cu-O) × *d*(Mn-O) × cos(**135°**)

→ *d*(Cu-Mn)^2^ = *d*(Cu-O)^2^ + *d*(Mn-O)^2^ + 2*d*(Cu-O) × *d*(Mn-O) × cos(**45°**)

We can use the fitting results of EXAFS of biogenic Mn oxide and sorption samples (Table 1), and then, get the maximum Cu-Mn distance is **3.98** Å when we use the maximum Mn-O distance (2.350 Å). The minimum value of Cu-Mn distance is when the plane of Cu atom and the two sharing O atoms parallels to the equatorial plane of the MnO_6_, which looks like the CuO_6_ and MnO_6_ share 3 O atoms and the Mn-O-Cu angle is 90°. And then, we can get a minimum theoretical value of Cu-Mn distance **2.73 Å** when we use the minimum Mn-O distance (1.903 Å).

**References**

1. Zhang ZJ, Yin H, Tan WF, Koopal LK, Zheng LR, Feng XH, Liu F: **Zn sorption to biogenic bixbyite**-**like Mn_2_O_3_ produced by *Bacillus* CUA isolated from soil: XAFS study with constraints on sorption mechanism**. *Chem Geol* 2014, **389**: 82–90.
2. Pan G, Qin YW, Li XL, Hu TD, Wu ZY, Xie YN: **EXAFS studies on adsorption**-**desorption reversibility manganese oxide**-**water interfaces I. Irreversible adsorption of zinc onto manganite (γ**-**MnOOH)**. *J Colloid interface Sci* 2004, **271**: 28–34.
